# Chief Executive Officer Tenacity and Employee Intrapreneurial Behavior: The Mediating Role of Corporate Social Responsibility

**DOI:** 10.3389/fpsyg.2022.829567

**Published:** 2022-03-08

**Authors:** Zheng Huang

**Affiliations:** School of Business, Renmin University of China, Beijing, China

**Keywords:** CEO tenacity, employee intrapreneurial behavior, corporate social responsibility, upper echelons theory, social information processing theory

## Abstract

Chief executive officer (CEO) tenacity plays an important role in corporate entrepreneurial activity. However, much less is known about its impact on employee intrapreneurship. Drawing from social information processing theory and upper echelons theory, this article examines the hitherto unexplored nexus between CEO tenacity and employee intrapreneurship, as well as the mediating role of corporate social responsibility (CSR). Quantitative data were collected through a survey administered to 294 employees working in different sectors that engage in CSR activities in China. Data analysis was performed using hierarchical regression method through Stata 16.0. It was found that CEO tenacity was significantly positively correlated with employee strategic renewal behavior (*β* = 0.523, *p* < 0.001) and employee venture behavior (*β* = 0.510, p < 0.001). The positive correlation between CEO tenacity and CSR was also significant (*β* = 0.578, *p* < 0.001). Besides, CSR partially mediated the relationship between CEO tenacity and employee strategic renewal behavior (40.0%) or employee venture behavior (50.2%). This study extends research on CEO tenacity, CSR, or employee intrapreneurial behavior by providing a better understanding of the direct effects of CEO tenacity on employee intrapreneurial behavior and CSR. From the perspective of cross-fertilization between psychology and management, this study establishes the interface role of CSR by elucidating the intrinsic mechanism of CEOs with high levels of tenacity to stimulate employee intrapreneurial behavior through CSR.

## Introduction

A key aspect of entrepreneurship is chief executive officer (CEO) tenacity (Baum and Locke, 2004; De Clercq and Belausteguigoitia, 2017a; Zeng and Ouyang, 2020). Recent years have witnessed a growing interest in the significance of CEO tenacity in entrepreneurial activity (Murnieks et al., 2016; Tadajewski and Jones, 2017; van Scotter and Garg, 2019). However, previous researchers have not extended this to employee intrapreneurship. CEO tenacity is meaningful for both CEO themselves and the employees, in that they may enhance employees’ problem-focused voice (De Clercq and Belausteguigoitia, 2017b) and contribute to their occupational innovation (Safavi and Bouzari, 2019; Li et al., 2020b). In the context of COVID-19 and anti-globalization, companies are recognizing the importance of building core competitive advantage through intrapreneurship. Meanwhile, more recent evidence suggests that employee intrapreneurship, including employee strategic renewal behavior employee venture behavior, helps organizations improve performance and build competitiveness (Criado-Gomis et al., 2018; Gawke et al., 2019; Pellegrini et al., 2019; Luu, 2020). The antecedents driving employee intrapreneurship as a focus of internal strategic innovation have attracted much attention at present (Huang et al., 2021), such as employees’ personality (Mahmoud et al., 2020), organizational support (Chouchane et al., 2021), and spiritual leadership (Usman et al., 2021), but empirical studies on CEO psychological traits need to be further developed. Therefore, it is novel for this study to examine whether and how CEO tenacity can have an impact on employee intrapreneurship.

Social information processing (SIP) theory is a theory in interpersonal communication that has been used in the organizational management literature to explain how leaders influence the attitudes and behaviors of their followers. SIP theory states that individual perceptions, attitudes, and behaviors are shaped by informational cues in the environment, which may be derived from institutional and cultural factors, the values and expectations of others, and so on (Salancik and Pfeffer, 1978; Bhave et al., 2010). Scholars thus have devoted considerable attention to the study of CEOs’ influence on employees through social information processing effects, and in particular to the study of leadership or CEOs’ characteristics that successfully fuel employee creativity or corporate innovation (Zhang and Wang, 2020; Ali et al., 2021). To sum up, SIP theory provides a theoretical link between CEO tenacity and employee intrapreneurship.

According to upper echelons theory, CEOs play an important role in the strategic choices of enterprises. Aguinis (2011) argued that CSR can be seen as a strategic decision that is the responsibility of top management and proposed a “triple bottom line” definition of CSR: “context-specific organizational actions and policies that take into account stakeholders’ expectations and the triple bottom line of economic, social, and environmental performance.” It is an investment decision, a business model, and a way to fulfill social obligations in the long term. The profit maximization principle is often not satisfied in CSR decisions, and shareholders may reject CSR decisions because they do not see immediate financial returns. Thus, there is opposition in many aspects of CSR decisions for CEOs. Much of what we know about CSR from this theory perspective is based on analyses performed with CEO characteristics or experiences, such as CEO’s ability (Yuan et al., 2019), bachelor’s degree, career experience, and gender (Manner, 2010). But most of this literature used observable demographics as proxies for psychological characteristics. Lawrence (1997) pointed out that directly examining the underlying psychological attributes of CEOs would enhance the validity of studies. Therefore, recent studies have begun to directly focus on the impact of CEO psychological attributes on CSR (Davidson et al., 2018; Tang et al., 2018). However, CEO tenacity, as a trait to maintain the high level of personal energy needed to achieve goals in the face of opposition or other difficult situations (Baum and Locke, 2004), is an understudied perspective for helping CEOs overcome opposition and resource dilemmas in CSR decisions. In addition, combining with SIP theory, this paper argues that CSR creates an interface linking CEO tenacity and employee intrapreneurial behavior. Specifically, CEO tenacity forms situational cues in the work environment by enhancing CSR, bringing positive feelings and resource security to employees, and therefore can promote their intrapreneurial behavior.

## Theories and Hypotheses

### CEO Tenacity and Employee Intrapreneurial Behavior

The entrepreneur-CEO tenacity is “a trait that involves sustaining goal-directed action and energy even when faced with obstacles” (Baum and Locke, 2004). It reflects how CEOs regulate their personal energy when achieving long-term work goals related to the development and organizational change of the enterprise. Entrepreneurs who have high levels of tenacity are more likely to be steadfast in their efforts and focus on entrepreneurial success (Kuratko, 2016), since they are more entrepreneurial and more capable of developing valuable skills (Baum et al., 2001). Moreover, CEO tenacity is significantly and positively related with venture growth (Baum and Locke, 2004), entrepreneurial persistence (Van Scotter and Garg, 2019), and investor support (Murnieks et al., 2016). In entrepreneurial activities, entrepreneurs bear the opportunity cost of other options than developing and managing an enterprise, liquidity premium of time and capital, market risk of uncertainty, environmental risk of technological development, and other hindrances, and will repeatedly encounter many adversities; tenacity stimulates the energy level of entrepreneurs and enhances their ability to overcome obstacles, therefore increasing the chances of success of entrepreneurial ventures (Markman and Baron, 2003). Although most studies have explored CEO tenacity’s significance in corporate entrepreneurial activity (Markman et al., 2005; Murnieks et al., 2016; Tadajewski and Jones, 2017), few have focused on its impact on CSR activity and employee intrapreneurship.

Employee intrapreneurial behavior is defined as “a specific type of agentic, strategic work behavior comprising employee venture behavior and strategic renewal behavior,” which is “more likely to be pursued by individuals who have a positive perception of opportunities in the work environment” (Gawke et al., 2019). According to Giang and Dung (2021), employee strategic renewal behavior (ESRB) is a behavior in which employees strive to find solutions to update operation and business strategies and improve company performance; employee venture behavior (EVB) is an attempt by employees to redesign the company’s products or services, as well as to develop new markets and create new business within their organizations. Both dimensions of employee intrapreneurial behavior are an employee-initiated creative and risk-taking activity within organizations, which has some similarity to entrepreneurial behavior of entrepreneurs. Notably, employees may benefit from CSR (May et al., 2021; Vătămănescu et al., 2021) and contribute to sustainable organizational performance (Suler et al., 2021) when doing intrapreneurship.

SIP theory theorizes that individuals’ beliefs, attitudes, and behaviors are not given but are products of individuals’ attempts to make sense of information processing activities in their environment (Salancik and Pfeffer, 1978), providing a theoretical perspective for understanding how CEO tenacity affects employee intrapreneurial behavior through work environment directly. Organizational members, such as employees, develop perceptions of significant others in a shared work environment, which in turn shapes matching work behaviors (Chadwick and Raver, 2015). In the work environment, the CEO, as a strategist and spiritual leader, is an important source of social information for employees, who shape their attitudes and behaviors by following their leaders (Bass, 1990; Weierter, 1997). Li et al. (2020b) identified the significance of entrepreneur’s psychological capital on employees’ innovation behavior and the underlying mechanisms of leader-member exchange (LMX) relationship, and CEO tenacity provides employees with just such a positive perception of their leader and then their work environment.

According to SIP theory, employees assess and evaluate their work environment to determine if they are confident enough to go into intrapreneurship. Furthermore, CEOs with high levels of tenacity not only succeed in their own entrepreneurial activities (Baum and Locke, 2004), but also provide positive situational cues to inspire their employees to take up intrapreneurship. CEO tenacity is considered the best profitable psychological trait to overcome adversity and succeed in business (Tadajewski and Jones, 2017; De Clercq and Belausteguigoitia, 2017a). Thus, employees’ perception of CEO tenacity impacts directly on their motivation, energy, and faith to continue their strategic renewal behavior and venture behavior. Therefore, this study posits that as:

*H1a:* There is a positive relationship between CEO tenacity and employee strategic renewal behavior.

*H1b:* There is a positive relationship between CEO tenacity and employee venture behavior.

### CEO Tenacity and CSR

Upper echelons theory states that CEO characteristics determine strategic choices and performance consequences of enterprises (Hambrick and Mason, 1984). As a key strategic choice, CSR is the integration of social welfare, business models, and investment patterns (Barnett, 2007; Zhao et al., 2021). The business objective of a company is to maximize shareholder wealth. In contrast, CSR activities seek to build or maintain a corporate reputation image in the long term (Zhao et al., 2021), which is conducive to the long-term value goals and sustainable organizational performance (Luu, 2020; May et al., 2021; Vătămănescu et al., 2021). However, this may be at odds with the short-term profit goals of the firm. Therefore, engagement in CSR activities requires CEOs to overcome resistance from all sides and persevere.

CEO tenacity is the tendency to persevere and endure in challenging and obstacle-filled environments (Stoltz, 1997; Markman et al., 2005) and can shape his or her personal success in leadership (Locke, 2000), vision communication, new resource skills, and self-efficacy (Baum and Locke, 2004). It can fuel CEO’s long-term goal achievement (Duckworth et al., 2007). Also, it is related to CEO’s risk selection and cross-period risk selection (Zeng and Ouyang, 2020). Meanwhile, CSR also requires significant financial investment and does not see tangible returns in the short term, which can be detrimental to shareholders’ interests. CEOs may encounter opposition when deciding on CSR choice. However, when CEOs have a high level of tenacity, they have the energy needed for the ongoing process of CSR activities and can also endure the loss of short-term benefits. They continuously overcome obstacles and difficulties to make the business better. Therefore, the CEOs’ level of tenacity is important when they make CSR decisions. Hence, this study proposes that as:

*H2:* There is a positive relationship between CEO tenacity and CSR.

### Mediating Effect of CSR

The role of CEO tenacity in stimulating employee strategic renewal behavior and employee venture behavior may be shaped by CSR. Although CSR activities are made by decision makers considering the interests of three parties, they reflect the social mission of organizations. The triad of economic, social, and environmental social responsibility covers both internal and external organization citizenship behaviors, whether it is fulfilling responsibilities externally in terms of social and environmental aspects or internally in terms of optimizing employment conditions for employees, showing an ethical organization image (Turker, 2008; Zhang, 2021).

Within the firm, CSR can be translated into soft systems related to the firm’s procedures, structure, and culture that provide productive resources for employees to work (Waddock and Graves, 1997). With the development of technology, new manufacturing techniques (Coatney and Poliak, 2020), artificial intelligence (Cunningham, 2021; Galbraith and Podhorska, 2021) or other automated production systems (Harrower, 2019; Suler et al., 2021) can enhance the sustainable performance of the company. However, the psychological recognition is undoubtedly more important for organization reform and employees innovation (Gao et al., 2020; Woo and Kang, 2021). When employees perceive that their organization is a responsible member of society, it helps them to identify with their work and, accordingly, employees are more willing to work with the organization to accomplish reform and maintain the sense of identity and self-esteem that the organization brings to them. This is also a social information processing effect. Moreover, May et al. (2021) demonstrated that the CSR initiative of an organization will improve the trust relationship between the organization and its employees, and then motivate employees to adjust their green behaviors to meet the expectations of the organization.

When CEOs have high levels of tenacity, they overcome obstacles to engage in activities related to the long-term goals of enterprises, therefore engage in more CSR activities. The above theoretical analysis suggests that CSR may promote employees’ intrapreneurial behavior, and thus, CSR creates an interface where CEO tenacity exerts social information processing influence on employees. Simsek et al. (2017) defines the interface as a “key explanatory mechanism through which influence is conveyed, perceptions and impressions are formed, and by which the attributes, aspirations, and activities of strategic leaders permeate the wider organization and beyond.” Thus, the organizational-level mechanism that underlies the relationship between CEO tenacity and employee intrapreneurial behavior is CSR. CEO tenacity translates the leader’s intrinsic mental strength into a resource guarantee through CSR; employees are easily influenced by the CEO’s social information processing through CSR. CEO tenacity enhances CSR, consistent with the fact that CSR performance reflects the CEO’s personal energy, and CSR leads to employee intrapreneurial behavior. Thus, this study proposes the hypotheses:

*H3a:* CSR mediates the relationship between CEO tenacity and employee strategic renewal behavior.

*H3b:* CSR mediates the relationship between CEO tenacity and employee venture behavior.

**Figure 1:**
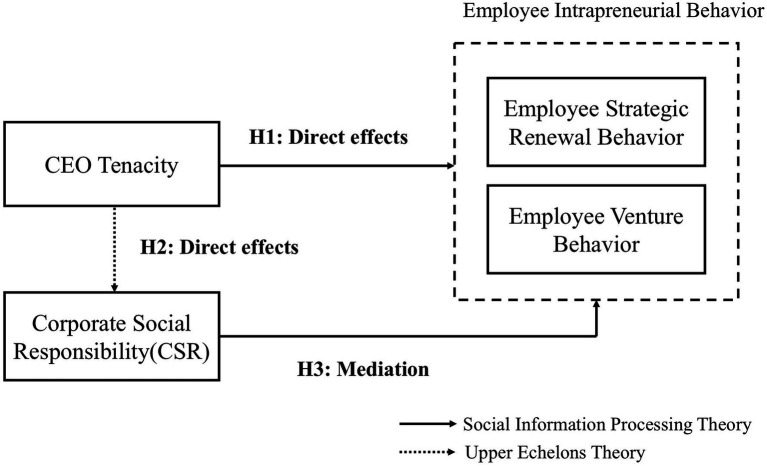
Theoretical model.

The proposed model of the study is shown in Figure 1.

## Materials and Methods

### Sample and Data Collection

This study used convenient sampling techniques to collect data from employees working in different sectors that engage in CSR activities in China. The researcher distributed online questionnaires in career forums and through personal relations. All respondents were asked to have work experience in enterprises. A total of 301 questionnaires were distributed, and this study excluded seven samples with significantly smaller response times. Finally, 294 complete and valid questionnaires were retained. Parameter values of Table 1 show the sample diversity.

**Table 1:** Sample characteristics.

Demographic characteristics	Frequency	%
**Gender**
1. Male (Gender = 1)	153	52.04
2. Female (Gender = 0)	141	47.96
**Age**
1. Less than 25 years (Age = 1)	30	10.20
2. 25–34 years (Age = 2)	111	37.76
3. 34–44 years (Age = 3)	89	30.27
4. More than 44 years (Age = 4)	64	21.77
**Level of education**
1. No bachelor degree (Edu = 1)	49	16.67
2. Hold a bachelor degree (Edu = 2)	146	49.66
3. Hold a master degree or higher (Edu = 3)	99	33.67
**Number of years in an organization**
1. Less than 1 year (Tenure = 1)	73	24.83
2. 1–3 years (Tenure = 2)	74	25.17
3. 3–5 years (Tenure = 3)	50	17.01
4. More than 5 years (Tenure = 4)	97	32.99
**Management level**
1. Ordinary employee (Manag = 1)	161	54.76
2. First-line manager (Manag =2)	83	28.23
3. Middle-level manager or higher (Manag = 3)	50	17.01

### Measurements

All items of main four constructs used 7-point Likert scales, ranging from 1 = strongly disagree to 7 = strongly agree (See Appendix).

#### CEO Tenacity (Tenacity)

I measured CEO tenacity with three items used in previous research (Baum and Locke, 2004; De Clercq and Belausteguigoitia, 2017a). Sample items included “The CEO of my organization continues to work hard on tasks even when others oppose him or her” and “I can think of many times when the CEO of my organization persisted with work when others quit.” The Cronbach’s alpha for this measure was 0.893.

#### Corporate Social Responsibility

I measured CSR using an 18-item scale adapted by Pasricha et al. (2018). Sample items included “My organization encourages employee participation in decision making process” and “My organization engages in program for water and/or waste recycling/reuse.” The Cronbach’s alpha for this measure was 0.975.

#### Employee Intrapreneurial Behavior (ESRB and EVB)

I measured employee intrapreneurial behavior using a scale developed by Gawke et al. (2019). There are 15 items of two dimensions in this scale: employee strategic renewal behavior (eight items) and employee venture behavior (seven items). Sample items included “I attempt actions to transform the existing product/service for the organization” and “I attempt actions to achieve emerging markets for the firm.” The Cronbach’s alpha for employee strategic renewal behavior was 0.956 and for employee venture behavior was 0.941.

#### Control Variables

Reference to previous literature (Luu, 2020), five demographic characteristics (age, gender, education level, organizational tenure, and management level) were controlled in this study to account for alternative explanations of employee intrapreneurial behavior.

## Results

### Confirmatory Factor Analyses

Before the tests of the hypotheses, I did a series of confirmatory factor analyses (CFAs) to rule out potential common method variance and verify the distinctiveness of the four constructs. Table 2 shows the results of CFAs.

**Table 2:** Results of confirmatory factor analysis for the main measures.

Model	Factor	χ^2^	df	χ^2^/df	IFI	TLI	CFI	RMSEA
One-factor model	Tenacity + CSR + ESRB + EVB	3809.726	594	6.414	0.690	0.670	0.689	0.136
Two-factor model	Tenacity +CSR; ESRB + EVB	2068.058	593	3.487	0.858	0.848	0.857	0.092
Three-factor model	Tenacity; CSR; ESRB + EVB	1740.295	591	2.945	0.889	0.881	0.889	0.081
Four-factor model	Tenacity; CSR; ESRB; EVB	1019.620	588	1.734	0.958	0.955	0.958	0.050

The one-factor model is a model combining all items of the four constructs into a single factor, and the fit indexes (*χ*^2^/df = 6.414 > 3, IFI = 0.690 < 0.9, TLI = 0.670 < 0.9, CFI = 0.689 < 0.9, RSMEA = 0.136 > 0.1) performed poorly, indicating that there is no homology error problem and good discriminant validity. Meanwhile, only the fit indexes for the four-factor model met the corresponding statistical criteria (*χ*^2^/df = 1.734 < 3, IFI = 0.958 > 0.9, TLI = 0.955 > 0.9, CFI = 0.958 > 0.9, RSMEA = 0.050 < 0.1) and performed better than both the two-factor (*χ*^2^/df = 3.487 > 3, IFI = 0.858 < 0.9, TLI = 0.848 < 0.9, CFI = 0.857 < 0.9, RSMEA = 0.092 < 0.1) and three-factor (*χ*^2^/df = 2.945 < 3, IFI = 0.889 < 0.9, TLI = 0.881 < 0.9, CFI = 0.889 < 0.9, RSMEA = 0.081 < 0.1) models.

### Descriptive Statistics

Table 3 shows the means, standard deviations, and correlations between variables for the main variables. Tenacity and ESRB (*r* = 0.542, *p* < 0.001), tenacity and EVB (*r* = 0.549, *p* < 0.001), tenacity and CSR (*r* = 0.633, *p* < 0.001), ESRB and CSR (*r* = 0.558, *p* < 0.001), and EVB and CSR (*r* = 0.618, *p* < 0.001) all showed significant positive correlations. These results preliminary supported the plausibility of this study’s hypotheses.

**Table 3:** Means, standard deviation (SD), and correlation.

S. no	Variables	Mean	SD	1	2	3	4
1.	Tenacity	4.741	1.623	1			
2.	ESRB	4.604	1.571	0.542^***^	1		
3.	EVB	4.433	1.496	0.549^***^	0.691^***^	1	
4.	CSR	4.550	1.503	0.633^***^	0.558^***^	0.618^***^	1

*N* = 294

*** *p* < 0.001 (two tailed).

### Regression Analysis

This study used hierarchical multiple regressions to test the hypotheses, that is, the control variables, the explanatory variable (CEO tenacity), and mediating variable (CSR) were sequentially put into the models. All models in this study were tested for multicollinearity before regression analysis, it was found that the VIF value of each model was less than 2, and the tolerance was greater than 0.1, so there was no serious multicollinearity problem.

Table 4 provides multiple regression results. The explanatory variable in Models1-3 was employee strategic renewal behavior, where Model 1 was the baseline model with control variables only. Model 2 was used to test the relationship between CEO tenacity and employee strategic renewal behavior (H1a). Model 3 was used to test the mediating role of CSR between CEO tenacity and employee strategic renewal behavior (H3a). The explanatory variable in Models 4–6 was employee venture behavior, where model 4 was the baseline model with control variables only. Model 5 was used to test the relationship between CEO tenacity and employee venture behavior (H2a). Model 6 was used to test the mediating role of CSR between CEO tenacity and employee venture behavior (H3b). Models 7–8 were used to test the mediating role of CSR, where Model 7 was the baseline model including only control variables, and Model 8 was used to test the relationship between CEO tenacity and CSR.

**Table 4:** Multiple regression results.

Variables	ESRB			EVB			CSR	
	Model 1	Model 2	Model 3	Model 4	Model 5	Model 6	Model 7	Model 8
Gender	0.381^*^	0.370^*^	0.289	0.279	0.269	0.169	0.236	0.225
Age	0.008	0.046	0.014	0.095	0.132	0.092	0.047	0.089
Tenure	−0.111	−0.083	−0.062	−0.056	−0.029	−0.003	−0.089	−0.058
Edu	−0.034	−0.042	−0.065	−0.054	−0.061	−0.089	0.071	0.063
Manag	−0.123	−0.002	0.050	−0.122	−0.004	0.060	−0.277^*^	−0.143
Tenacity		0.523^***^	0.314^***^		0.510^***^	0.254^***^		0.578^***^
CSR			0.362^***^			0.443^***^		
*R* ^2^	0.027	0.313	0.383	0.019	0.319	0.434	0.035	0.417
△*R*^2^		0.286^***^	0.070^***^		0.300^***^	0.115^***^		0.382^***^

*N* = 294;

* *p* < 0.05,

*** *p* < 0.001 (two tailed).

Combining the results of Model 1 and Model 2, it could be seen that the amount of variance explained by CEO tenacity on ESRB was 28.6% after controlling for the effects of other variables, which indicated that CEO tenacity had a significant explanatory role on ESRB. In support of my prediction H1a, Model 2 revealed that CEO tenacity related positively to ESRB (*β* = 0.523, *p* < 0.001), in strong support of H1a.

Combining the results of Model 4 and Model 5, it could be seen that the amount of variance explained by CEO tenacity on EVB was 30.0% after controlling for the effects of other variables, which indicated that CEO tenacity had a significant explanatory role on EVB. In support of my prediction H1b, Model 4 revealed that CEO tenacity related positively to EVB (*β* = 0.510, *p* < 0.001), in strong support of H1b.

Model 8 added explanatory variables to the baseline Model 7, and the variance explained by CEO tenacity reached 38.2%, indicating the important explanatory role of CEO tenacity on CSR. Also, Model 8 revealed that CEO tenacity relates positively to CSR (*β* = 0.578, *p* < 0.001), in strong support of H2.

In this paper, I used Baron and Kenny (1986) approach in a series of multiple regressions to test for the mediating effect. I had already shown the positive relationship between CEO tenacity and CSR, ESRB, and EVB in the above section. Model 3, a regression of ESRB on both CEO tenacity and CSR, showed that CSR related positively to ESRB (*β* = 0.362, *p* < 0.001). Compared with Model 2, the impact of CEO Tenacity on ESRB has decreased (from 0.523 to 0.314). The mediating effect volume for CSR between CEO tenacity and ESRB is 40.0% (0.578^*^0.362/0.523). Similarly, Model 6, a regression of EVB on both CEO tenacity and CSR, showed that CSR related positively to EVB (*β* = 0.443, *p* < 0.001). Compared with Model 5, the impact of CEO Tenacity on EVB has decreased (from 0.510 to 0.254). Also, the mediating effect volume for CSR between CEO tenacity and EVB is 50.2% (0.578^*^0.443/0.510). Consistent with H3a and H3b, these results suggested that CSR mediated the relationship between CEO tenacity and ESRB or EVB.

## Discussion

Prior studies have established the significance of CEO tenacity for their personal career (Baum et al., 2001; Markman and Baron, 2003; Zeng and Ouyang, 2020) or organizational development (Baum and Locke, 2004; Murnieks et al., 2016; van Scotter and Garg, 2019) in entrepreneurial activity. Up to now, far too little attention has been paid to its impact on employee intrapreneurial behavior. This study adds to our understanding of the association of CEO tenacity with employee intrapreneurial behavior and CSR, and the underlying theoretical mechanisms of this association. Specifically, it explores the direct impact of CEO tenacity on employee intrapreneurial behavior and CSR, as well as the mediating effects of CSR between CEO tenacity and employee intrapreneurial behavior. In the empirical design, employee intrapreneurial behavior is split into two dimensions, ESRB and EVB. The theoretical foundation of the study is grounded on the social information processing theory and upper echelons theory. The study results provided empirical support for all hypotheses depicted in the proposed theoretical model. In contrast to previous studies, which have focused on a single CEO dimension or a single employee dimension, the current study introduces CSR as a construct of corporate organizational institutional factors that can provide an interface for CEO tenacity to have an impact on employee intrapreneurial behavior with social information processing implications and connects the two theories, creating a cross-fertilization perspective of psychology and management.

In consistency with SIP theory, one important finding is the positive relationship between CEO tenacity and ESRB (*β* = 0.523, *p* < 0.001), as well as CEO tenacity and EVB (*β* = 0.510, *p* < 0.001). This result indicates that the direct effect of CEO tenacity on employee intrapreneurial behavior is significant and has a greater effect on employee strategic renewal behavior than on employee venture behavior (0.523 > 0.510). It suggests that this trait, which excels in helping entrepreneurs in their entrepreneurial activities, also motivates employee intrapreneurial behavior. This somewhat accords with previous evidence, which showed that entrepreneur’s psychological capital was significantly positively correlated with employee innovation behavior (Gao et al., 2020; Li et al., 2020b). They are based on LMX theory or goal theory, which suggests that positive psychological traits of CEOs can strengthen the exchange relationships of organizational members or influence employees’ task goals. Based on SIP theory, this paper provides a new theoretical perspective to investigate how CEO psychological traits affect employee behavior.

Drawing on upper echelons theory, another important finding of this paper is the positive relationship between CEO tenacity and CSR (*β* = 0.578, p < 0.001). This result reveals that the direct effect of CEO tenacity on CSR. Moreover, this is consistent with previous studies linking CEO characteristics and CSR (Manner, 2010; Yuan et al., 2019). Specifically, their analysis using easily observable extrinsic demographics empirically supports the view that CEOs have a significant influence on CSR decisions and practices. Extending this, this paper introduces CEO tenacity in psychology field based on Lawrence (1997)’s suggestion to strengthen the validity of these studies. CEO psychology will become a new trend in CSR research (Li et al., 2020a).

Importantly, the most striking finding from this paper is that CSR serves as the mediator in the linkage of CEO tenacity and employee intrapreneurial behavior. The mediated effect volumes of CSR between CEO tenacity and ESRB and EVB are 40.0 and 50.2%, respectively. This result establishes that CSR can be used as an interface for linking the CEO and employees. Meanwhile, it offers initial corroboration of the critical role of CSR in social influence processing. In accordance with this result, previous studies have demonstrated the importance of CSR in improving corporate sustainability performance and promoting organizational development (Crișan-Mitra et al., 2020; May et al., 2021; Vătămănescu et al., 2021). Besides, the study extends understanding of the antecedents and consequences of CSR (Pasricha et al., 2018; Memon et al., 2021; Shen et al., 2021; Zhao et al., 2021). Furthermore, it is very interesting that the mediating role of CSR connects upper echelons theory and SIP theory.

## Theoretical Implications

This study may have several theoretical implications. First, this study extends the tenacity literature by finding its positive impact on employee intrapreneurial behavior. Researchers have acknowledged the key role of tenacity for individual entrepreneurial behavior (Kuratko, 2016) and organizational entrepreneurial success (Van Scotter and Garg, 2019). Baum and Locke (2004) noted that “there are other indicators of performance (personal satisfaction, survival, innovation, intangible assets) that must be studied.” This paper finds a positive relationship between CEO tenacity and CSR, employee intrapreneurial behavior. Both CSR and employee intrapreneurial behavior are important manifestations of effective organizational management, expanding the organizational context in which tenacity function as well as providing positive evidence for CEO tenacity.

Additionally, the current study contributes to the debate on antecedents driving employee intrapreneurial behavior. Based on social information processing theory, this study investigates the direct and indirect ways in which CEO tenacity affects employee intrapreneurship. Huang et al. (2021) constructed a framework for the literature on employee intrapreneurship and considered individual enablers, organizational enablers, and facilitating mechanisms. According to them, CSR may be an organizational-level element that, based on the previous discussion, may form a supportive structure that drives employee intrapreneurship. Then, CEO tenacity is relevant to all three elements, because it influences employees’ attitudes and judgments at the individual level through the social information processing process, and it may have an impact on resource availability and culture at the organizational level through CSR. Its direct impact on employee intrapreneurship and its indirect impact through CSR complements the five incentives proposed by Huang et al. (2021).

Finally, this paper contributes to CSR research by investigating CSR from the perspective of cross-fertilization between psychology and management. Moreover, this paper explicates the role of CSR in bridging the interface between CEO and employee relationships by examining the mediation of CSR between CEO tenacity and employee intrapreneurial behavior. Most of the previous studies have considered the antecedents or consequences within the organization of CSR but have not linked them together to form a logical chain (Pasricha et al., 2018; Giang and Dung, 2021; Memon et al., 2021; Zhao et al., 2021). Eventually, this paper will help us understand the possible mediating role of CSR as more than a performance-driven antecedent (Luu, 2020; May et al., 2021) or a consequence of CEOs’ decisions (Manner, 2010; Yuan et al., 2019).

## Practical Implications

The findings provide several practical implications. First, organizations can select, develop, and promote CEOs with high levels of tenacity based on their CSR strategies. Tenacity is a personal trait that helps CEOs maintain goal orientation and provides sustained personal energy for CEOs in the face of adversity, so I argue that this trait can also help CEOs in non-profit maximizing CSR decisions to overcome opposition and thus enhance CSR. Second, employees will engage in more intrapreneurial behavior when they feel that their CEO has high levels of tenacity and that their organization can deliver on CSR. In the context of COVID-19 and anti-globalization, it is imperative to stimulate intrapreneurship among employees as companies urgently need to build core competitive advantages to survive. This paper provides a “CEO-CSR-employee” path for strategic internal innovation in organizations. Third, CSR provides a linking interface between the CEO and employees within the organization, providing a positive rationale for the implementation of CSR decision. The CEO, as the strategic and spiritual leader of the enterprise, may lack sufficient communication opportunities with bottom-level employees. However, CEO tenacity will be reflected in the outcome of the CSR decisions of the enterprise, thus promoting the intrapreneurial behavior of employees.

## Limitations and Future Research Directions

Although this study attempts to clarify the influence mechanism of CEO tenacity on employee intrapreneurial behavior through CSR, due to the complexity of the research mechanism and the limitations of the research methodology, there are three limitations of this study and some issues that need to be further deepened in future studies. First, the respondents of this study are targeted at employees of Chinese enterprises, which may limit the generalizability of the findings. Therefore, future research could extend this paper’s model to other countries to assess the generalizability of the findings. Second, given the inherent limitations of the questionnaire method, future research could use a case study approach that looks at the specific impact of CEO personal traits on CSR and how it affects employees through the work environment. Finally, this paper examines the employees’ perceived CEO tenacity and CSR levels from their perspective, and future research could examine them from the CEO perspective.

## Conclusion

Putting in a nutshell, this empirical study has addressed questions of whether and how CEO tenacity can have an impact on employee intrapreneurship. Based on SIP theory, it proposes that CEOs with tenacity influence employee intrapreneurial behavior through social information processing, and the positive association of CEO with ESRB (*β* = 0.523, *p* < 0.001) and EVB (*β* = 0.510, *p* < 0.001) supports this hypothesis. According to upper echelons theory, tenacity can help CEOs overcome opposition in implementing CSR decisions, and the positive relationship between CEO tenacity and CSR (*β* = 0.578, *p* < 0.001) supports this hypothesis. One of the most significant findings from this study is that CSR partially mediated the relationship between CEO tenacity and employee strategic renewal behavior (40.0%) or employee venture behavior (50.2%). This implies that CSR can be seen as an interface for CEOs with tenacity to stimulate employee intrapreneurial behavior and connects upper echelons theory and SIP theory. This study hopes that these findings will serve as a catalyst for future research on the relationship between CEO psychological traits and employee behavior and prompt scholars to focus on the interface role of CSR.

## Data Availability Statement

The raw data supporting the conclusions of this article will be made available by the author, without undue reservation.

## Ethics Statement

Ethical review and approval were not required for the study on human participants in accordance with the local legislation and institutional requirements. The questionnaire for this study was completed by employees of companies in China, and the questions were about the company and its leaders, as detailed in the appendix, and did not involve issues of personal privacy, physical health, or other rights of the participants. In accordance with Chinese legal and institutional requirements, no ethical approval was required for the questionnaires conducted in this study. The patients/participants provided their written informed consent to participate in this study. Prior to data collection, the participants received confirmation of informed consent. Thus, the informed consent of the participants was implied through survey completion.

## Author Contributions

The author confirms being the sole contributor of this work and has approved it for publication.

## Funding

This work was supported by the Outstanding Innovative Talents Cultivation Funded Programs 2021 of Renmin University of China.

## Conflict of Interest

The author declares that the research was conducted in the absence of any commercial or financial relationships that could be construed as a potential conflict of interest.

## Publisher’s Note

All claims expressed in this article are solely those of the authors and do not necessarily represent those of their affiliated organizations, or those of the publisher, the editors and the reviewers. Any product that may be evaluated in this article, or claim that may be made by its manufacturer, is not guaranteed or endorsed by the publisher.

## Appendix

*CEO Tenacity*

The CEO of my organization continues to work hard on tasks even when others oppose him or her.
I can think of many times when the CEO of my organization persisted with work when others quit.
The CEO of my organization works harder than most people I know.

*Corporate Social Responsibility (CSR)*

My organization works with government officials to protect its interest.
My organization adopts a long-term perspective in decision making in order to guarantee sufficient cash flow and produce a persistent superior return to shareholders/owners.
My organization differentiates products/processes by marketing of their social and environmental performance.
My organization uses certification on quality aspects, e.g., ISO 9000.
My organization encourages employee participation in decision making process.
My organization encourages creation of good work-life balance and family friendly employment.
My organization invests in people, e.g., training and employee development.
My organization provides equal opportunities in workplace, e.g., employs disabled people and/or promotes women to senior management positions.
My organization engages in improving employee health and safety.
My organization engages in philanthropic activities, e.g., charitable donation.
My organization sponsors local community initiatives.
My organization considers interests of stakeholders in investment decisions by creating a formal social dialogue.
My organization engages in periodic natural environment audits.
My organization engages in environmental training for employees.
My organization uses filters and controls on emissions and discharges.
My organization engages in program for water and/or waste recycling/reuse.
My organization engages in increasing energy efficiency.
My organization uses certifications on environmental aspects, e.g., ISO 14000.

*Employee Strategic Renewal Behavior*

I attempt actions to transform the existing product/service for the organization.
I attempt activities that change the firm structure.
I attempt efforts to transform the working processes for the firm.
I contribute ideas for strategic renewal for my organization.
I undertake activities to realize the change in my organization.
I conceive innovative ideas of working for my company.
I exploit in-depth knowledge in the industry to innovate in my organization.
I exploit opportunities in the labor market or society to renew my organization.

*Employee Venture Behavior*

I attempt actions to establish structural subdivisions.
I attempt actions to achieve emerging markets for the firm.
I attempt to establish agencies externally for the firm.
I formulate new service procedures for the firm.
I attempt to establish start-ups within the bounds of the firm.
I conceptualize alternative products for my firm.
I actively establish new collaborations with experts outside of my own profession.
